# Identification of a novel FBN1 mutation in a Chinese family with isolated ectopia lentis

**Published:** 2011-12-29

**Authors:** Chen Liang, Wei Fan, Sisi Wu, Yi Liu

**Affiliations:** 1 Department Of Ophthalmology, West-China Hospital, Chengdu, China; 2 West-China Hospital, The State Key Laboratory of Biotherapy, Sichuan University Chengdu, China

## Abstract

**Purpose:**

To identify the genetic defect in a Chinese family with autosomal dominant inherited ectopia lentis.

**Methods:**

twenty-one family members, including seven patients underwent general physical and fully ophthalmic examinations. Genomic DNA was extracted from leukocytes of venous blood of these individuals in the family. Polymerase chain reaction (PCR) amplification and direct sequencing of all 65 coding exons of the fibrillin-1 gene (*FBN1*) were analyzed.

**Results:**

Mutation screening in FBN1 identified a T>C transition at nucleotide position c,1759 leading to substitution of Cysteine for Arginine at codon 587 (C587R). This nucleotide substitution was not seen in any unaffected member of the family.

**Conclusions:**

We detected a novel mutation in *FBN1*. Our result expands the mutation spectrum of FBN1 and help in the study of the molecular pathogenesis of Marfan syndrome and Marfan-related diseases.

## Introduction

Ectopia lentis (EL) refers to a displacement or malposition of lens from its normal location, in which the zonular filaments are stretched or discontinued [1]. In the majority of cases, ectopia lentis occurs as one symptom of systemic disease. Most cases of ectopia lentis are associated with Marfan syndrome, an autosomal dominant inherited disorder of connective tissue with prominent manifestations in cardiovascular, skeletal, and ocular systems [2].

A defect in the fibrillin-1 gene (*FBN1*) has been demonstrated to be associated with autosomal dominant inherit ectopia lentis [3,4]. *FBN1*, located on chromosome15q21, is approximately 235 kb in size. *FBN1* encodes fibrillin-1, a secreted 350-kDa glycoprotein known to be a major component of the elastin associated 10 to 12nm microfibrils, which is the major structure element in the suspensory ligaments of the lens. Fibrillin-1 is mainly composed of three types of repeated motifs. The first one is cysteine-rich epidermal growth factor (EGF)-like domain. There are 47 such modules, and 43 of them have a calcium binding consensus sequence(cbEGF-like). The second one is transforming growth factor beta-1 binding protein-like module (TGF beta1-BP-like module), which contains eight cysteine residues. This kind of motif is found seven times in FBN1. The third one is a hybid of EGF-like and TGF betal-like module, which occurs twice [5]. The disorders caused by FBN1 mutations are classified as Marfan syndrome and type 1 fibrillinopathies, which include Marchesani syndrome (MASS), isolated ectopia lentis, isolated skeletal features of MFS, and thoracic aortic aneurysms [6]. The identified mutations are distributed throughout *FBN1* and the correlation of genotype-phenotype is not clear yet.

In this study, we analyzed a Chinese family with EL and detected a novel heterozygous mutation in *FBN1*. In the family, the mutation cosegregated in the patients and was not observed in any of the unaffected family members.

## Methods

### Patients and clinic data

We came across a four-generation family affected with bilateral ectopia lentis at West China Hospital, Sichuan Province, China. The family history revealed nine affected members in four generations of which two were deceased and the youngest affect patient is twenty-three years old. We recruited 21 family members and we characterized the individuals in the family by ocular and physical examinations after obtaining informed consent from all the participants.

### DNA sequencing

Genomic DNA from 21 family members was extracted from 5 ml of blood by standard protocol. Candidate gene regions at 15q known to be linked with ectopia lentis were analyzed. All 65 exons of *FBN1* and their splice-junctions were amplified by RCR using the primers shown in Appendix 1.The primers 11, 41, 51, 52 and PCR conditions were designed as previous described [7]. The amplified PCR products were sequenced using ABI PRISM 3.0 Genetic Analyzer (ABI Applied Biosystems, Shanghai, China). The sequence results were analyzed by Chromas 2 (sequences view software).

## Results

### Clinical findings

**Figure 1:**
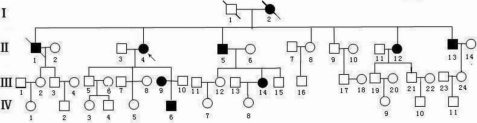
The pedigree of the family (the patient above the arrow is the proband). Squares and circles indicate males and females, respectively, and the darkened symbols represent the affected members. The patient above the arrow is the proband.

The pedigree of the family is shown in Figure 1. Bilateral lens ectopia was detected in seven patients and none of them displayed any abnormalities in the cardiovascular system and skeletal system. Patients include: II:4, II:5, II:13, II:14, III:9, III:14, and IV:6 (Table 1). Individual I:1 and II:1 died many years ago and both of them had poor vision acuity according to the description of their family members, but no related medical records were available.

**Table 1:** Clinic details of the patients in the family.

**Patients**	**II:4**	**II:5**	**II:12**	**II:13**	**III:9**	**III:14**	**IV:6**
Age	66	69	54	52	44	40	23
Gender	female	male	female	male	female	female	male
Ectopia lentis	+	+	+	+	+	+	+
Myopia	+	+	+	+	+	+	-
Abnormally flat cornea	-	-	-	-	-	-	-
Strabismus	-	-	+	+	-	-	-
Glaucoma	-	-	-	-	-	-	-
Retina detachment	-	-	-	-	-	-	-
Height(cm)	158	170	157	168	163	162	180
Arm span(cm)	157	172	157	169	165	161	183
AS/H(normal<1.5)	0.99	1.01	1	1.01	1.01	0.99	1.02
cardiovascular system	-	-	-	-	-	-	-
skeletal system	-	-	-	-	-	-	-

### Mutation findings

**Figure 2:**
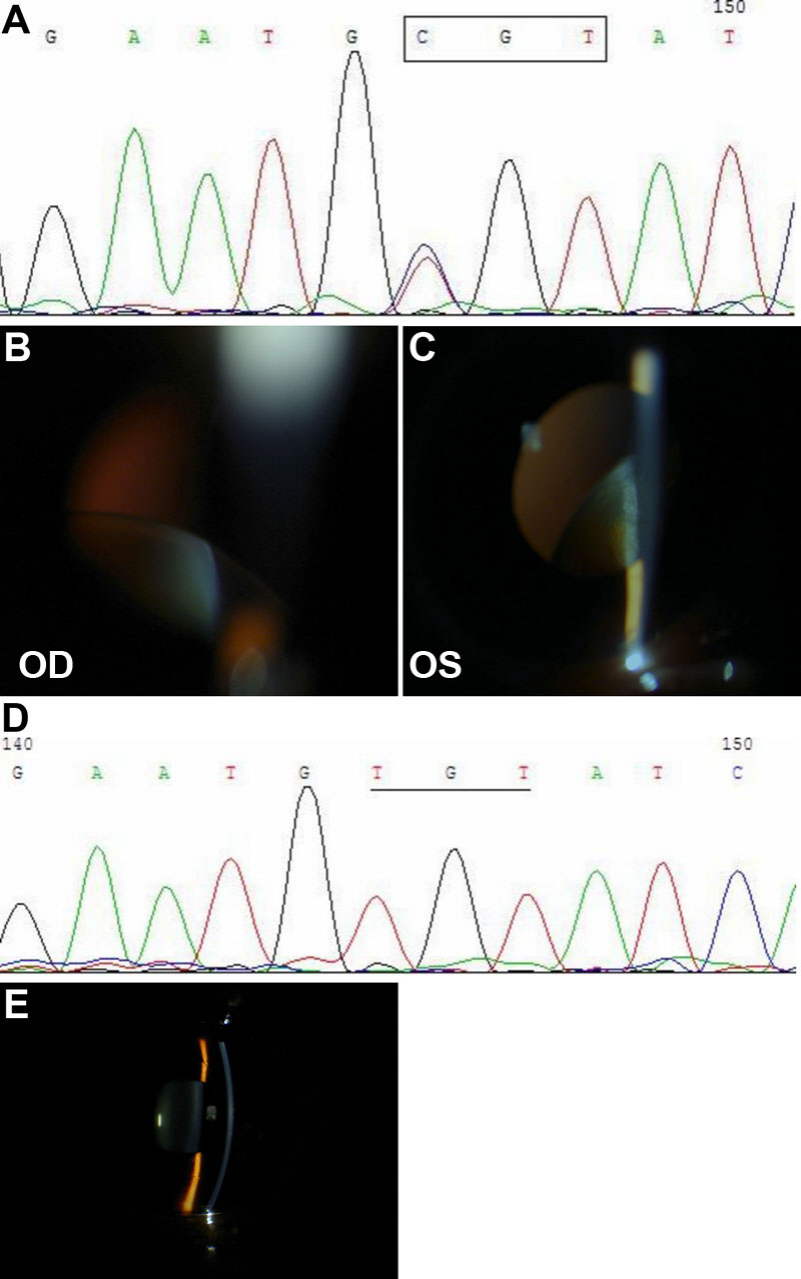
The mutation and images of the family. **A**: DNA sequence result shows a heterozygous T>C transversion in patient II:12. **B**: The right eye image of  patient(II:12). **C**: The left eye image of patient(II: 12). **D**: Normal sequence result of unaffected family member (III:3). **D**: The image of unaffected family member (III:3).

**Figure 3:**
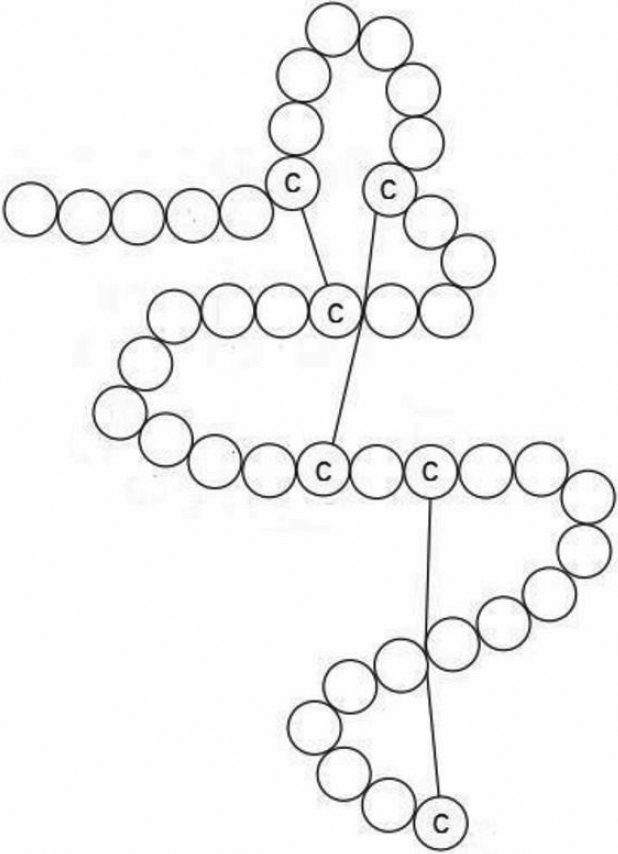
the structure of a cbEGF-like domain. The wild-type cbEGF-like domain which contains six conserved cysteine residues that are paired to form disulphides in a characteristic manner (1 to 3, 2 to 4,and 5 to 6).

After direct sequencing of *FBN1* in the family members, a missense mutation in exon 15 was discovered in seven patients and there were no mutations in unaffected family members (Figure 2). To the best of our knowledge, the mutation C587R has not been previously reported. This genetic variation alters a wild-type cysteine residue to a arginine residue in the 5th cbEGF-like dominant which contain six conserved cysteine residues that are paired to form disulphide in characteristic manner (1 to 3, 2 to 4, and 5 to 6; Figure 3).

## Discussion

In the present study, we have examined a Chinese family with isolated ectopia lentis and described a novel heterozygous mutation in *FBN1* (c.1759), leading to substitution of Cysteine for Arginine at codon 587. The substitution of one of the six conserved cysteines in the 5th cbEGF-like dominant probably causes domain misfolding.

We analyzed the patients from this study and from previous studies to evaluate the genotype-phenotype correlations of patients with mutation of cysteine. Schrijver et al. [8] described that premature termination codon (PTC) mutations appeared to be associated with more severe skeletal findings, whereas the cysteine substitution was associated with significantly greater incidence of ectopia lentis. Rommel et al. [9] analyzed *FBN1* in 116 patients with Marfan syndrome, showing a significantly lower incidence of ectopia lentis in patients whose mutation leading to PTC or missense mutation without cysteine involvement in FBN1,as compared to patients whose mutations involved a cysteine substitution. The interesting result is that the correlation of cysteine substitution with ectopia lentis.

### Possible pathogenesis

Fibrillin monomers polymerize to 10–12 nm microfibrils which associate with mature fibers to form the elastic fiber. The elastic fiber is essential for the function of resilient tissues, such as the aorta, the lung, and the skin. However, the microfibrils not associated with elastin were observed in ciliary zonule. And the microfibrils in ciliary zonule provide structural anchorage in non-elastic tissues [10]. In the examination of slit lamp microscope, we found the ciliary zonule of our patients could be seen in aqueous fluid with one end failing to anchor at the lens. We speculate that the mutation may have deleterious effects on the anchorage function of ciliary zonule, but may not effect the conformation of fibrillin or elastin fiber and the affected patients appear isolated ectopia lentis without abnormalities in the cardiovascular and skeletal system. The correct cysteine localization and disulfide bonding appears to play an important role in the anchorage function of the suspensory ligaments.

Since the first mutation of *FBN1* was identified in MFS patients in 1991, currently more than 1,200 *FBN1* mutations have been reported [4,11]. Most of them are missense mutations, and others are nonsense mutations, splice defects, or deletions. But the correlation of phenotype-genotype has not been built yet. Our data further expand the mutation spectrum of *FBN1* and contributes to the study of molecular pathogenesis of MFS and Marfan-related disorders. Further work involving animal model research and other ethnic groups might clarify the role that different mutations play in the pathogenesis of this condition.

## Appendix 1.

The list of primers for exons. To access the data, click or select the words “Appendix 1.” This will initiate the download of a compressed (pdf) archive that contains the file.
